# Synchronous visual analysis and editing of RNA sequence and secondary structure alignments using 4SALE

**DOI:** 10.1186/1756-0500-1-91

**Published:** 2008-10-14

**Authors:** Philipp N Seibel, Tobias Müller, Thomas Dandekar, Matthias Wolf

**Affiliations:** 1 Department of Bioinformatics, Biocenter, University of Würzburg, Am Hubland, Würzburg, Germany

## Abstract

**Background:**

The function of a noncoding RNA sequence is mainly determined by its secondary structure and therefore a family of noncoding RNA sequences is much more conserved on the structural level than on the sequence level. Understanding the function of noncoding RNA sequence families requires two things: a hand-crafted or hand-improved alignment and detailed analyses of the secondary structures. There are several tools available that help performing these tasks, but all of them are specialized and focus on only one aspect, editing the alignment or plotting the secondary structure. The problem is both these tasks need to be performed simultaneously.

**Findings:**

4SALE is designed to handle sequence and secondary structure information of RNAs synchronously. By including a complete new method of simultaneous visualization and editing RNA sequences and secondary structure information, 4SALE enables to improve and understand RNA sequence and secondary structure evolution much more easily.

**Conclusion:**

4SALE is a step further for simultaneously handling RNA sequence and secondary structure information. It provides a complete new way of visual monitoring different structural aspects, while editing the alignment. The software is freely available and distributed from its website at

## Background

Sequence alignments represent a starting point for almost every sequence based analysis. Analysing RNA sequences requires in addition to take information about their secondary structures into account. Therefore several methods like lara [1], MARNA [2], RNAforester [3] or Dynalign [4] were proposed in recent years to align RNA sequences taking into consideration their secondary structures. Since many downstream sequence analysis methods are based on the information represented by alignments, building correct alignments is challenging as both sequences and structures have to be taken into account.

A common task is to manualy check and improve the alignment before using it for further analysis. Programs like 4SALE [5], SCARNA [6], ConStruct [7], DCSE [8] or RALEE [9] were built to help manually editing RNA sequences and secondary structure alignments. These tools provide solid edit functionality, but in case of large complex alignments it is often hard to find the exact positions that require manual improvements. Visualizations can help to identify those misaligned regions. Conservation bars which are used in well known tools like ClustalX [10] and sequence logos [11] are the most common methods to visualize sequence alignments. But both these methods have the disadvantage that every single column needs to be checked manually. Although the methods can be used also in the context of sequence and secondary structure alignments, they are not specifically designed to take advantage of the secondary structure information. Sequence logos [11] were extended to RNA structure logos [12] and 3D sequence logos of RNA alignments [13] to also include structure information, but still fail to provide an easy understanding and compact visualization of the structural information contained in the alignment. A different approach of visualizing RNA alignment information is given with the S2S framework [14], which interconnects an RNA sequence alignment with a consensus secondary and the 3D tertiary structure visualization. While this approach can help to get a good understanding of the common structural information within the RNA sequence alignment, it can not handle individual secondary structure information. Therefore it is not capable of visualizing the structural differences within a sequence family.

A common practise to analyze at secondary structure information is to plot the structure in 2D. Several algorithms for a 2D secondary structure layout were proposed: Bruccoleri layout [15], loopdloop layout [16], vector based layout [17] or efficient RNA drawing [18]. These algorithms were implemented in tools like the Vienna Package [19], loopdloop [16], RNAViz [20], RNAMovies [21] or JViz.RNA [22] to provide a compact visualization of single secondary structures. StructureLab [23] provides more advanced interactive visualization of data derived from different folding algorithms. In addition, it has several facilities to visualize and compare data that are derived from multiple solutions for one sequence or multiple solutions from several sequences. RNAMovies [21] was the first program to provide a solution to visualize the differences of many secondary structure alternatives for one RNA sequence by animating between the 2D plot of every single structure alternative. 4SALE takes all these advantages of plotting single instances of secondary structures as well as the visualization of differences between structures and combines it with the main concept of 4SALE: synchronicity. This means every single edit operation or selection is synchronized with a new 2D plot of either the complete alignment as consensus visualization or a selected single secondary structure. The 2D plot animates between all changes made in the alignment in a similar way as RNAMovies [21] does. This makes it easy to understand what the changes in the alignment imply for the secondary structures.

## Implementation

The new visualization capabilities in 4SALE are built on top of prefuse [24], an interactive information visualization toolkit for JAVA. Prefuse provides a rich set of features for visualization and animation capabilities. We used existing RNA secondary structure layout algorithms and integrated them with prefuse to enable interactive secondary structure visualization. The structure viewer is integrated like any other component in 4SALE working with RNA sequence and secondary structure information, this allows seamless interaction between all other components like the alignment window or the window showing the compensatory base changes (CBC table).

## Results

### Structure Viewer

**Figure 1:**
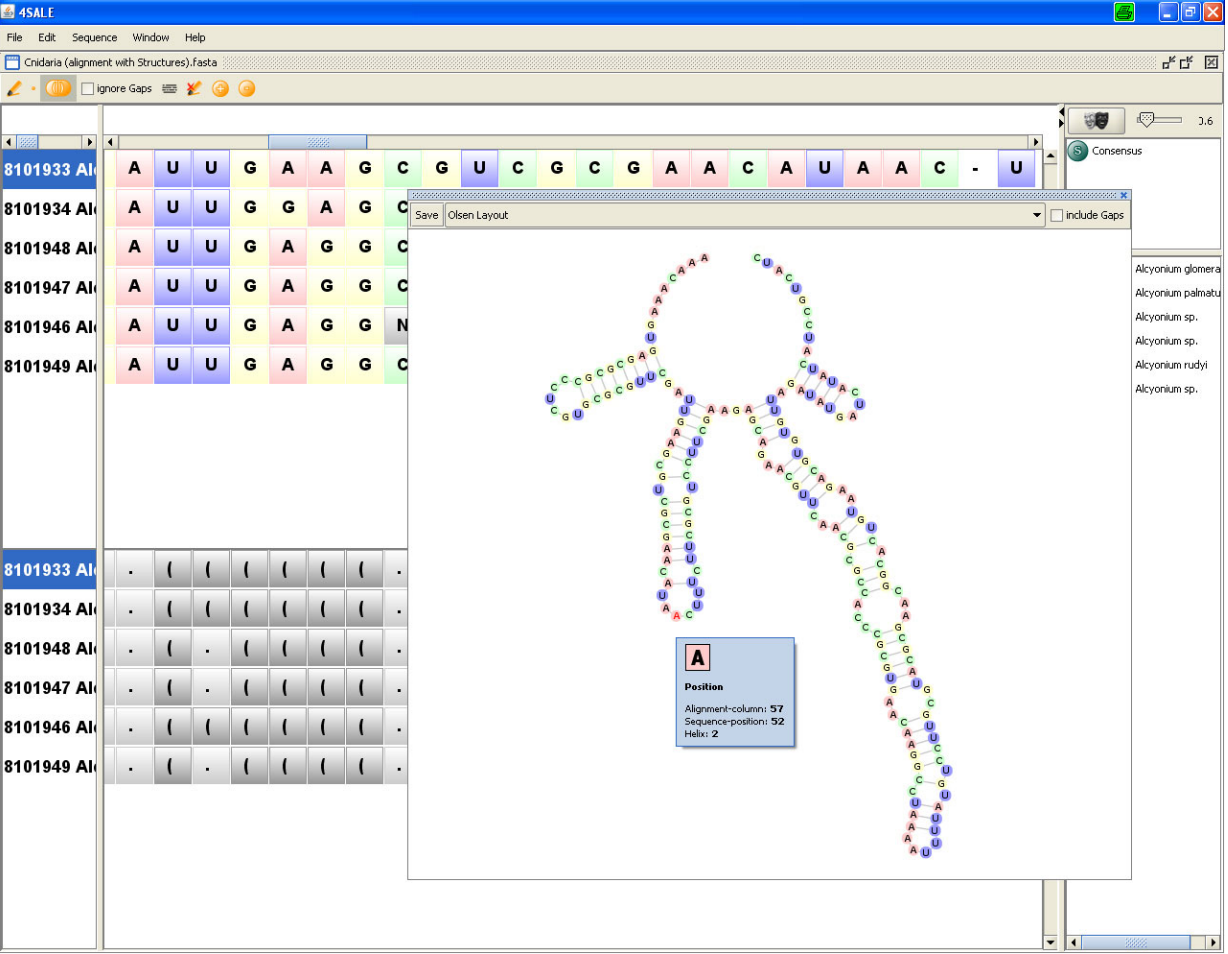
**Structure viewer in single structure mode**. By selecting a sequence in the alignment window its colored secondary structure is shown in Olsen layout. Optionally, this structure can be viewed in the Bruccoleri layout as well. Additionally, gaps within the multiple sequence alignment can be projected onto the secondary structure by checkmarking the "include gaps" box. The current visualization can be saved as image in different formats (svg, jpg and png).

The new structure viewer (Fig. 1) allows visualization of 2D plots of the secondary structures within the sequence and secondary structure alignment. By selecting sequences in the alignment window the corresponding secondary structures are loaded into the viewer. Visualization of single and multiple sequences are supported. The two most popular layout algorithms of Bruccoleri [15] and Olsen (as implemented in loopdloop [16]) are available. The Bruccoleri layout is based on a per helix alignment, while the Olsen layout also prevents clashes of adjacent nucleotides within loops by allowing angles in one helix to resolve space limitations. The plot can optionally be drawn with gaps, which causes all gap positions to be handled as dots (unpaired positions). If multiple sequences are selected, the consensus structure based on a user-defined conservation value is plotted by default. By unchecking the "conservation mode"-checkbox a single structure navigator is shown, which allows easy switching between selected sequences.

### Visualization of structural differences

Although the RNA secondary structures within the alignment can be categorized by the consensus structure, it is also important to look at the individual differences between the structures, especially if the alignment is not highly conserved. 4SALE provides animations between secondary structures similar to RNAMovies [21], which makes it easy to detect the differences. While RNAMovies [21] animates through the secondary structure landscape of one single RNA sequence by using the sequence positions as anchor points for the animation, we use the alignment columns to provide animation between structures of distinct RNA sequences, which therefore are not required to be of equal length.

### Synchronized selection

Since 4SALE synchronizes every action between all views on the same dataset, selections within the structure view automatically appear in the alignment window and vice versa. The selection is context sensitive, which means it is only applied to the current selected sequences. This is extremely helpful to highlight patterns that can be easily selected via the search function within the alignment window, within the structure view. One example might be the conserved UGGU motif 5' side to the apex of helix III in ITS2 [25].

### Monitoring structure information during editing

4SALEs live structure preview allows monitoring all structure related modifications on the sequence and secondary structure alignment. Blockwise editing in "edit alignment mode" modifies the consensus structure for example, these changes are shown as animations within the structure view, when "consensus mode" is activated. This feature easily helps to prevent misalignments. Editing secondary structures or manually adding secondary structures to loaded sequences was already supported by 4SALE, by activating the structure viewer it is now possible to view the current state of the secondary structure information. By selecting one sequence the construction process of this sequence is animated with every insertion/deletion of a bond in the alignment window. This method is also available for column based edit operations, in this case the current consensus structure is animated.

### Mapping alignment information to a consensus structure

**Figure 2:**
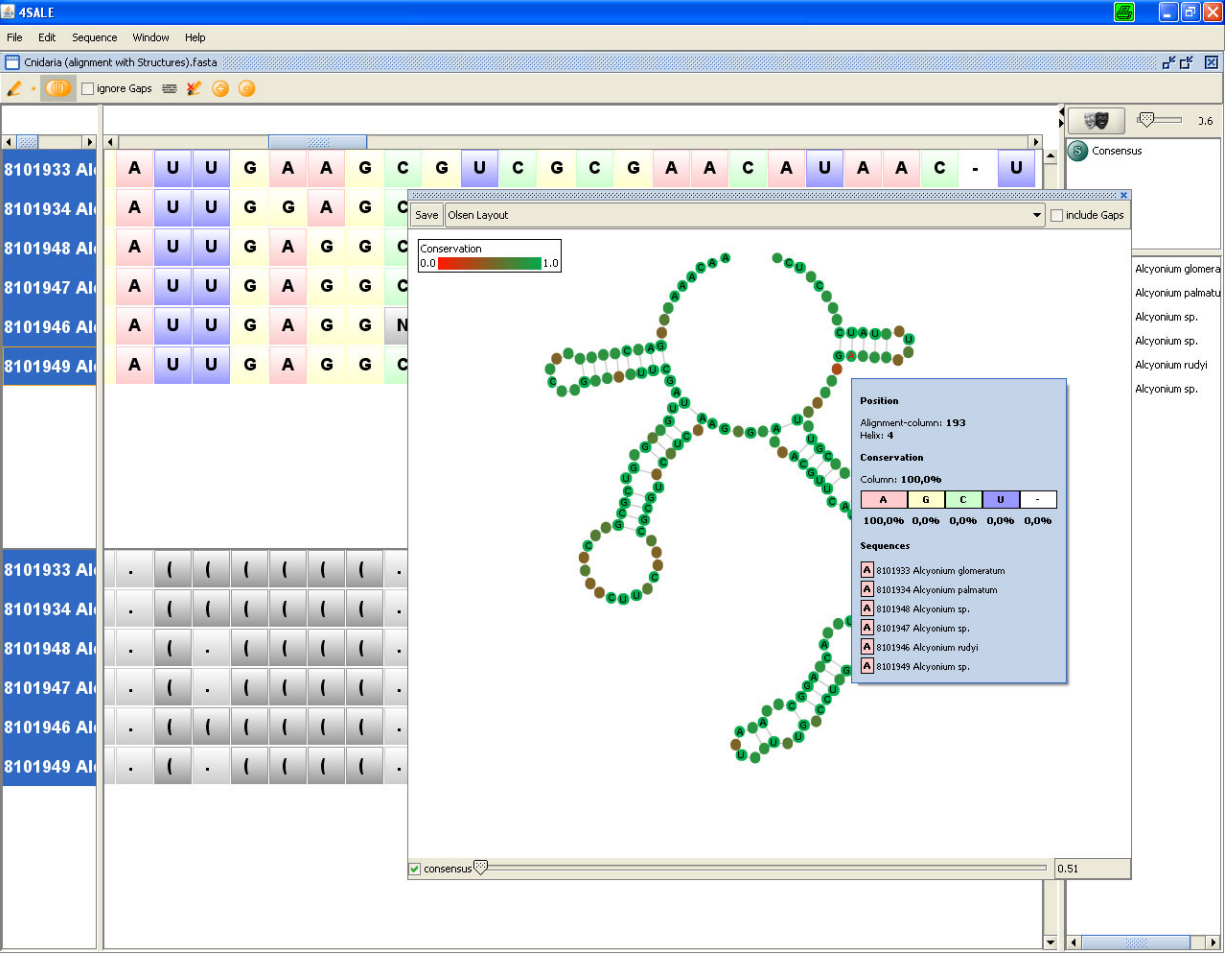
**Structure viewer in consensus mode**. The structure viewer enables a compact visualization of sequence and secondary structure alignments by plotting the consensus structure. Additional alignment information like conservation, gap information, composition, helix position and alignment column position is provided within the tooltip. A slider on the bottom of the winodw allows to choose the degree of secondary structure conservation. Sequence conservation is visualized via colorinterpolation between red (no conservation) and green (highly conserved).

4SALE provides a compact visualization of sequence and secondary structure alignments (Fig. 2) by plotting the consensus structure and labeling it with alignment information. The common structure information of all selected sequences is represented by the plotted consensus structure while the sequence information is visualized by a position wise colormap based on the degree of conservation within the current alignment column. Since every dot in the structure view represents a column in the alignment, the tooltip at this position contains specific information about this column, like nucleotide based conservation or which helix this position is in.

### Analyzing compensatory base changes (CBCs)

**Figure 3:**
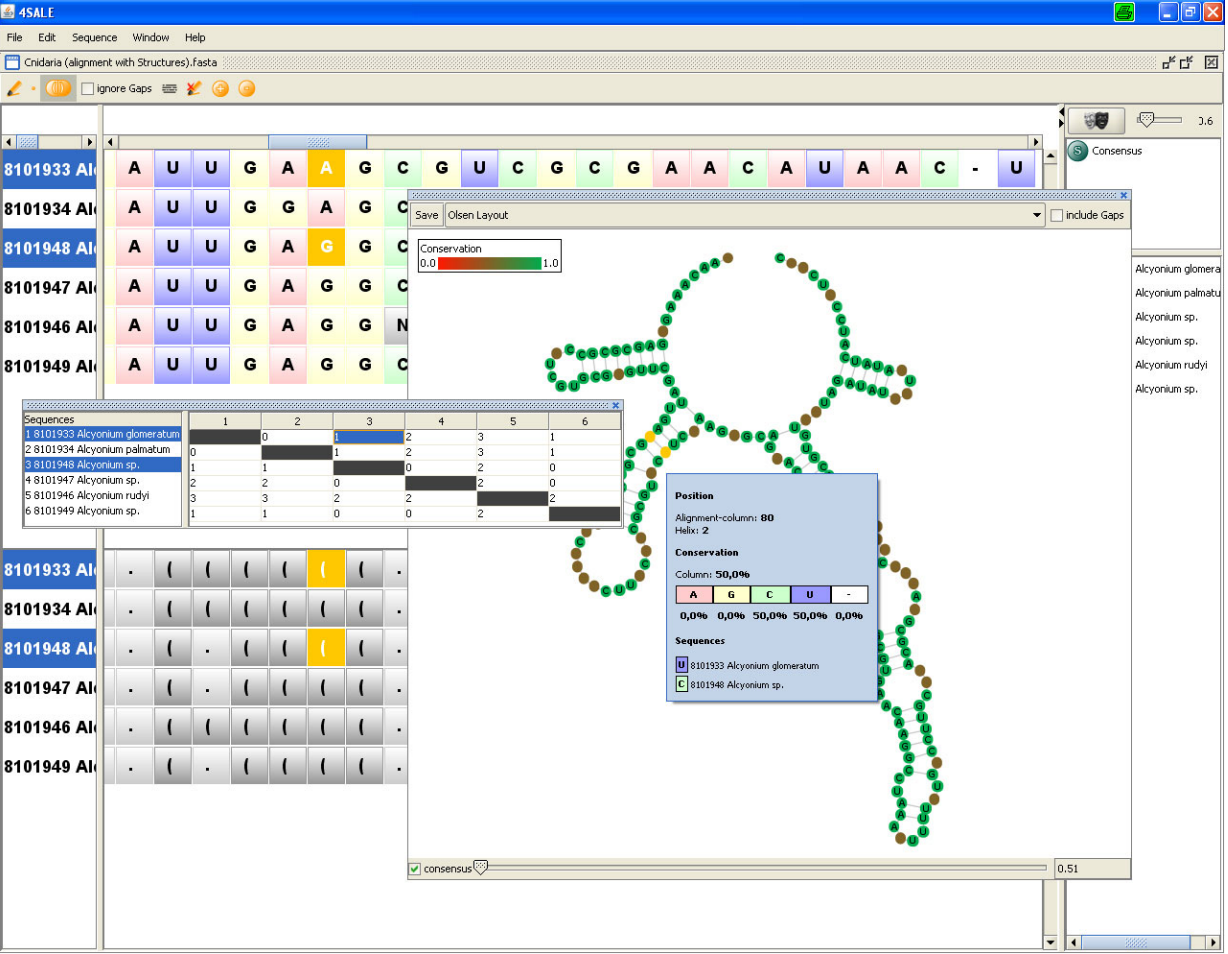
**CBC analysis using the structure viewer**. Due to synchronization between all 4SALE windows, marked CBCs in the CBC table are highlighted in the alignment and the structure window.

The synchronization of selected regions (Fig. 3) leads to a big improvement of the existing CBC analyzing capabilities of 4SALE. Corresponding base changes selected in the CBC table are now not only highlighted within the alignment window, but also within the structure viewer. This provides a much more intuitive view to understand CBCs beetween two RNA sequences. One can either look at the common structure element shared by both sequences, which is shown by default or activate the sequence navigator to animate between the two sequences.

### Saving secondary structure images

Secondary structure plots are often used to illustrate specific regions within RNA sequences. 4SALE supports exporting the current state of the structure viewer to a vector or pixel based image. By supporting Scalable Vector Graphics (SVG) it is possible to further manipulate, comment and label the plot without loosing quality.

### Other improvements

Besides the new visualization capabilities 4SALE also introduced some other new features that help working with sequence and secondary structure alignments. First we added the ability to modify sequence information, which was requested by many users. By placing the cursor in the sequence part of the sequence and secondary structure view while "edit alignment mode" is active, it is now possible to simply type the letters to be inserted or use backspace to remove parts of the sequence. When inserting new nucleotides 4SALE sets the corresponding structure part as nonbinding positions. When deleting nucleotides 4SALE checks whether binding regions are involved and automatically removes all related nucleotide pairings before performing the deletion. It is to be noted that due to the complexity of this operation, undo operation are currently only supported with gap insertions or deletions. However, for all other options, the undo operation is available.

4SALE also adds lara [1] integration, a tool for sequence and secondary structure alignments. This allows users to choose between the 4SALE alignment method or the lara alignment method [1]. The 4SALE alignment maps the sequence and secondary structure information of every single RNA sequence to one letter encoded sequences. The algorithm can be described as string alignment on a 12 letter alphabet comprised of the 4 nucleotides in three structural states (unpaired, paired left, paired right). Horizontal dependencies given by the nucleotide pairings are not modeled by this approach. To align the encoded sequences common alignment programs, like CLUSTAL W [26] with a suitable scoring matrix can be used [5]. In contrast to the faster 4SALE alignment method, lara [1] uses combinatorical optimization and respects horizontal dependencies.

Furthermore we added the possibility to export the one letter encoded sequence and secondary structure alignment used with the 4SALE alignment method. Together with the substitution matrix located in the matrix folder this output can be used to calculate phylogenetic trees considering both, sequence and secondary structure information, by using standard protein based phylogenetic tools, like ProfDist [27] or PHYLIP [28]. Note that the provided substitution matrix is calculated on sequences of the ITS2 database [29,30], so it should be used with caution with other RNA sequence families than ITS2.

## Discussion and conclusion

A number of RNA visualization methods cover either sequence or structure visualization. Here we introduce a novel method to visualize sequence and secondary structure alignments. By mapping information contained in RNA sequence and secondary structure alignments to a single 2D structure plot of the associated consensus structure, a compact and informative overview is presented. In contrast to so far used methods like sequence/structure logos or conservation bars, our method takes advantage of the structural information to collect all the information in one view. The previous column based visualization of alignments didn't scale very well with the length of the alignment. We adressed this problem by plotting the information in two dimensons instead of a linear representation. This not only prevents the user from scrolling through all columns of the alignment but also results in a very intuitive view of the secondary structure information contained.

With the tight integration of this new visualization technique into the existing editor, 4SALE moves a step further and provides a comfortable environment when working with RNA sequence and secondary structure information. The integrated viewer shows simultaneously structure and sequence information and enables visual monitoring structural aspects while editing the sequence-structure alignment. This joint view leads to a better understanding of sequence-structure evolution. Potential application of 4SALE includes evolutionary and phylogenetic studies, the comperative study of RNA molecules, and RNA design, e.g. in mutation studies.

The new features of 4SALE are demonstrated by a movie provided within the help of 4SALE at .

## Abbreviations 

CBC: compensatory base change; ITS2: internal transcribed spacer 2.

## Competing interests

The authors declare that they have no competing interests.

## Authors' contributions

MW conceived the study. PS elaborated the visualization concept. Architecture, implementation and graphical design by PS. MW, PS and TM drafted the manuscript. MW, TM, JS and TD participated in ms writing, study design and coordination. All authors read and approved the final version of the manuscript.
